# Study of the Urinary Ratio of 6 β-Hydroxycortisol/Cortisol as a Biomarker of CYP3A4 Activity in Egyptian Patients with Chronic Liver Diseases

**Published:** 2007-02-07

**Authors:** Ehab S. ELDesoky, Sherif I. Kamel, Ahlam M. Farghaly, Madiha Y. Bakheet, Mohsen A. Hedaya, Jean-Pascal Siest

**Affiliations:** 1 Pharmacology Dept, Faculty of Medicine, Assiut University, Assiut, Egypt; 2 Department of Tropical Medicine and Gastroenterology, Assiut University Hospital, Assiut, Egypt; 3 Department of Tropical Medicine and Gastroenterology, Assiut University Hospital, Assiut, Egypt; 4 Department of ClinicalPathology, Assiut University Hospital, Assiut, Egypt; 5 Department of ClinicalPharmacy, Faculty of Pharmacy, Tanta University, Tanta, Egypt; 6 Chairman, Managing Direction of STABILIGEN.jusqu’au/till 21/XII/2002: 32 rue Lionnois - B.P. 292. 54005 NANCY, Cédex – France

**Keywords:** 6 β-hydroxycortisol/cortisol ratio, CYP 3A4, hepatic cirrhosis

## Abstract

The urinary ratio of 6 β-hydroxycortisol/cortisol (6 β-OHC/C) as a biomarker of CYP3A4 metabolizing activity has been studied in Egyptian patients with chronic liver cirrhosis associated with previous hepatic Schistosomiasis infection to determine any possible alteration in enzyme activity. The ratio of 6-β OHC/C was determined in morning urine samples collected from 8:00 a.m. to 12:00 p.m. in healthy adults (n = 36) and patients with liver cirrhosis (n = 57). The median age for control was 27 years (range: 18–50 years) and 50 years (range: 27–75 years) for patients. 6 β-OHC was detected in urine by ELIZA kits (Stabiligen, France). Patients with liver cirrhosis were categorized according to Child Pugh Classification into Child B (n = 28) and Child C (n = 29) classes. Cholestasis was observed in 9/28 of Child B class and 8/29 of Child C class of patients. The control subjects showed gender-related difference in the urinary ratio of 6 β-OHC/C. A significant reduction (*P* < 0.001) in 6 β-OHC/C ratio was observed only in Child C patients in comparison with control subjects. Regression analysis showed a significant correlation (*P* < 0.05) between 6 β-OHC/C ratio and serum albumin. The influence of cholestasis on the urinary ratio of 6-β OHC/C was observed on cirrhotic patients of Child B class. In conclusion, patients with chronic liver cirrhosis might have a reduction of metabolizing activity of CYP3A4 enzymes which could be identified by measuring the urinary ratio of 6 β-OHC/C. This reduction is more apparent in severe liver injury (Child C class). Therefore, it is important to understand the metabolic fate of drugs metabolized by 3A4 enzymes in patients with liver cirrhosis to avoid drug accumulation that might lead to development of drug toxicity.

## Introduction

Cytochrome P450 enzymes (CYP450) are heme-containing monooxygenases responsible for the metabolism of numerous xenobiotics including therapeutic drugs, environmental chemicals, dietary constituents and endogenous substrates such as steroids and bile acids. They activate metabolic transformation of these compounds into water soluble and less toxic substances that are easily eliminated from the body (Eichelbaum and Burk, 2001; Furuta et al. 2003). However, these enzymes may also transform nontoxic chemicals into reactive intermediates that are likely toxic or carcinogenic (Rendic S and DiCarlo, 1997).

CYP3A is the most highly expressed subfamily of cytochrome P450 isozymes in humans and it includes the isoforms 3A4, 3A5, 3A7 and 3A43 (Shimada et al. 1994). The CYP3A subfamily plays an important role in the biotransformation of more than 50% of drugs in clinical use such as macrolide antibiotics, benzodiazepines, calcium channel blockers, immunosuppressive drugs and chemotherapeutic agents (Thummel and Wilkinson, 1998). CYP3A4 is the most abundant isoform expressed in liver and intestine with high interindividual variability in its protein level and catalytic activity (Guengerich, 1992; Ozdemir, 2000).

Several noninvasive in vivo probes for estimating the inter-patient variability of CYP3A4 activity have been reported and include the erythromycin breath test, the urinary dapsone recovery test, measurement of midazolam clearance (CL), and measurement of the ratio of endogenous urinary 6-beta-hydroxycortisol to free-cortisol (Hunt et al. 1992; Watkins et al. 1992; Kinirons et al. 1993; Thummel et al. 1994). Of all the probes tried, midazolam plasma clearance and the erythromycin breath test have been the most rigorously studied and appear to be the most reliable methods (Streetman et al. 2000; Goh et al. 2002).

Estimation of urinary ratio of 6 β-hydroxycortisol/cortisol (6 β-OHC/C) has been suggested as a simple non-invasive biochemical marker to determine the activity of CYP3A4 in human (Wilkinson, 1996; Galteau and Shamsa, 2003). Although the ratio does not always correlate with the disposition of CYP3A substrate drugs, including the typical phenotyping probe drugs such as [^14^C]-erythromycin and midazolam (Hunt et al. 1992; Watkins et al. 1992; Kinirons et al. 1993, 1999), but recent studies have described successful utilization of the urinary ratio 6 β-OHC/C as a measure of CYP3A induction (Saima et al. 2002; Uejima et al. 2002; ELDesoky et al. 2005; Yeo et al. 2006). Furthermore, the urinary ratio has also been studied in some disease conditions to elucidate a possible correlation of CYP3A activity with disease states such as hepatic disease, thyroid disorders and cancer (NG et al. 1996; Zheng et al. 2001; Szucs et al. 2003; Kishino et al. 2004; Hoshiro et al. 2006).

Liver cirrhosis due to chronic hepatitis B or hepatitis C viral infection is a common medical problem in Egypt. In many cases especially for hepatitis C (HCV) infected patients living in rural areas, it is accompanied by Schistosomal hepatic fibrosis due to previous Schistosomal parasitic infestation (Angelico et al. 1997). This combined viral and parasitic liver infection exhibits a unique clinical, virological, and histopathological pattern where the infective virus persists with high HCV RNA titers, the necroinflammatory and fibrosis scores in liver biopsies become higher and response to interferon therapy is poor (Pereira, 1995; Kamal et al. 2000). With progression of liver cirrhosis, both liver function and hepatic blood flow are impaired. So, the metabolizing capacity of liver microsomal enzymes including CYP3A has been expected to reduce (Shiffman et al. 1995; Bastein et al. 2000).

In the present study, we have estimated the urinary ratio of 6 β-hydroxycortisol/cortisol in spot urine samples of Egyptian patients with chronic liver cirrhosis due to hepatitis B or hepatitis C viral infection associated with previous schistosomal infection. The urinary ratio of 6 β-hydroxycortisol/cortisol was used as a simple non-invasive biomarker to assess CYP3A4 activity in these hepatic patients in comparison with Egyptian control subjects. The influence of cholestasis if present in association with the hepatic disease conditions on the ratio was also investigated. Furthermore, a possible correlation between the urinary ratio of 6 β-OHC/C and different biochemical parameters of liver function (e.g. serum albumin and transaminases) in cirrhotic liver was also studied.

## Material and Methods

### Patient and control subjects

Fifty seven patients with post-hepatitis liver cirrhosis due to hepatitis B or hepatitis C viral infection associated with past history of Shistosomal infection were selected for the study. Patients attended the Tropical Medicine and Gastroenterology Department, Assiut University Hospital, Assiut, Egypt for medical care. The diagnosis of liver cirrhosis was based on clinical manifestations of liver cell failure (e.g. ascites and jaundice), biochemical findings (e.g. hypoalbuminemia, hyperbilirubinemia, abnormal coagulation profile and elevation of liver enzymes) and ultrasonography. Endoscopy was carried out if needed. Serological tests for hepatitis B virus antigen (HBsAg) and hepatitis C virus antibodies (HCVAb) confirmed the previous viral hepatitis infection of the liver. The serological tests for hepatitis markers were done using Abbott AXSYM System. The method of assay is based on micro particle enzyme immunoassay (MEIA). Inclusion or not of cholestasis in association with liver cirrhosis was based on Clinical findings such as itching, jaundiced skin or sclera, deep dark urine and clay colored or white stool. Laboratory diagnosis of cholestasis included elevation of serum levels of both bilirubin and alkaline phosphatse enzyme [ALP] in parallel with elevation of serum gamma glutamyl transferase [GGT] enzyme. Also, abdominal ultrasonography and CT scan imaging studies helped to confirm the intrahepatic obstructive lesions that caused cholestasis.

Diagnosis of previous Schistosomal infection relied upon endemicity of Schistosomal infection in rural areas where selected patients were living, past history of Schistosomal infection and exposure of the patients to anti bilharzial drug therapy, and rectal biopsy. Abdominal sonography including the liver (for the presence of Schistosomal hepatic periportal fibrosis) was applied to confirm previous Schistosomal affection of the liver. No patient had clinically active Schistosomiasis. Patients had no history of alcohol intake but 15 of them were cigarette smokers. No drugs known to affect the activity of CYP3A4 were given to the patients (e.g. macrolide antibiotics, contraceptives, or antifungal agents of an azole structure). Table 1 demonstrates the demographic and relevant clinical data of selected patients.

Thirty six healthy subjects (26 males & 10 females) were included in the study as a control group with median age of 27 (range: 18–50 years). They were non-smokers and have no history of previous chronic illness and laboratory findings especially liver and kidney function tests were within normal. Also, they had negative history of receiving drugs known to affect CYP3A4 activity for at least two months before running of the study.

Liver function tests were done for all subjects (patients & control) in the Clinical Pathology laboratories at Assiut University Hospital, Assiut, Egypt. They included determination of serum albumin, bilirubin, alanine aminotransferase (ALT), aspartate aminotransferase (AST), and alkaline phosphatase. Also, serum creatinine and prothrombin time were estimated.

### Urine samples collection

In the morning of the day of the study, urine samples were collected as spot urine in the period from 08:00 a.m. to 12:00 a.m. Then, samples for each patient were divided into portions and kept frozen at −20°C without adding any preservatives till the time of assay.

### Assay methods

#### (i) Assay of urinary 6 β-hydroxycortisol (6 β-OHC)

Urinary concentrations of 6β-OHC were assayed using enzyme-linked immunoassay kits (ELISA technique) which were given as a gift from Stabiligen (54603, Villers-Les-Nancy-France). The mechanism of measurement by the kits was based on a competitive immune reaction where the competitive ligands in the kits were 6 β-hydroxycortisol conjugated to horseradish peroxidase and penicillinase enzymes respectively (Zhiri et al. 1986). The minimum detection limit (sensitivity) of 6β-OHC by Stabiligen kits was 50 pg/ml. The intra-assay precisions were 4% (C.V %) at a mean concentration of 697 pg/ml and 10% at a 69.9 pg/ml respectively. The inter-assay variations were 3% at a mean concentration of 731 pg/ml and 7.2% at a mean concentration of 72.1 pg/ml.

#### (ii) Assay of free cortisol in urine

Free cortisol was assayed in urine using DSL-2100 Active^©^ Cortisol coated-tube radio immunoassay kits (Diagnostic Systems Laboratories, Inc, Webster, Texas 77598-4217, U.S.A). The sensitivity of the assay was 0.3 μg/dl. The intra-assay precisions were 8.4% (C.V %) at a mean concentration of 5.0 μg/dl and 5.3% at 19.2 μg/dl respectively. The inter-assay variations were 9.1% at a mean concentration of 4.83 μg/dl and 8.9% at 19.18 μg/dl respectively.

## Statistical Analysis

The ratio of 6β-OHC/C was expressed as median values and non-parametric tests e.g. Mann-Whitney U test was used for comparison. Regression analysis and analysis of variance (ANOVA) were used to assess a possible correlation between urinary ratio of 6β-OHC/C and any of the demographic factors (e.g. age, gender, and smoking) or biochemical parameters (e.g. serum creatinine, albumin, bilirubin, alkaline phosphatase, etc) measured in cirrhotic patients. Statistical significance was considered at p < 0.05.

## Results

The influence of urine concentration on the urinary ratio of 6β-OHC/C in control subjects was assessed by studying the correlation between levels of urinary creatinine and corresponding ratio of 6β-OHC/C in urine samples. No significant relation was observed.

Assessment of urinary ratio of 6β-OHC/C in control subjects showed wide range (1–73 ng/ml) with significantly higher median values in males compared with female controls (Table 2).

Table 3 demonstrated the influence of liver cirrhosis on the values of urinary ratio of 6β-OHC/C. Child C but not Child B cirrhotic patients had a significant reduction (p < 0.001) in the urinary ratio of 6β-OHC/C compared with control subjects. Also, Child C patients had significantly lowered urinary values of 6β-OHC/C in comparison with Child B cirrhotic patients.

Table 4 showed a trend (but not significant) for urinary ratio of 6β-OHC/C in patients with Child B class of liver cirrhosis to elevate in the presence of cholestasis compared with control subjects. On the other hand, Child B patients with no cholestasis had a significant lowering of the ratio of 6β-OHC/C compared with control group. Regarding Child C class of patients, it seemed that the severe liver cell injury in this class of patients irrespective of the presence or absence of cholestasis was significantly affecting the urinary ratio of 6β-OHC/C in comparison with Child B and/or control subjects.

**Figure 1:**
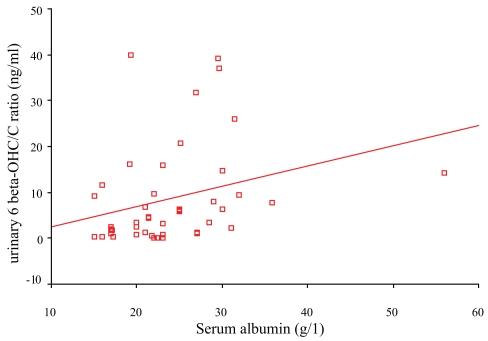
Relation between urinary 6 beta OHC/C ratio and serum albumin in cirrhotic patients (r = 0.4, P < 0.05).

Non-parametric correlation analysis revealed no significant influence of smoking, age, or creatinine clearance on the urinary ratio of 6β-OHC/C in patients with hepatic cirrhosis. Study of the relation between urinary ratio of 6β-OHC/C and different biochemical parameters of liver function demonstrated a significant correlation between the ratio and serum albumin (r = 0.4, p < 0.05) (Fig. 1). On the other hand, both ALT and prothrombin time showed a trend to negative but not significant correlation with the urinary ratio of 6β-OHC/C.

## Discussion

The present study showed gender-related difference in the activity of CYP3A4 among Egyptians (Table 2). Among the control group, females had lower values of the urinary ratio of 6β-OHC/C in comparison with males; a result that coincides with previously reported data among Egyptians (ELDesoky et al. 2005) and other populations (Lee, 1995; NG et al. 1996). However, other studies demonstrated higher values of the urinary ratio in females compared with males (Inagaki et al. 2002). Although lack of gender influence on the activity of the enzyme was also reported (McCune et al. 2001), some studies reported differences in the urinary ratio of 6β-OHC/C in the same sex but of different ethnicity (Lin et al. 1999). Therefore, the influence of gender on the activity of CYP3A4 is still an unresolved observation.

The wide range of the urinary ratio of 6β-OHC/C (1.0–73.0 ng/ml) in the control group is in agreement with other studies (Chen et al. 2004; Yin et al. 2004) which all reflect a wide interindividual variability in the urinary excretion of 6β-hydroxycortisol in different populations. Among the Egyptian controls studied, gender seems to play a role in this variability between males and females. However, the genetic component is likely to contribute significantly in the variability (Yin et al. 2004). Therefore, an expected variability in CYP3A4 gene expression among different populations should be considered (Hustert et al. 2001; Koch et al. 2002; Lin et al. 2002).

Child C class of patients where more deterioration of liver function was expected showed significant reduction in the urinary ratio of 6β-OHC/C in comparison with control subjects and Child B class of patients (Table 3). These results reflected possible reduction in the activity of CYP3A4 in patients with chronic liver cirrhosis to form 6 β-hydroxycortisol metabolite and the more the liver injury, the more the reducing effect on CYP3A4 activity. A previous study demonstrated similar reduction in the urinary ratio of 6β-OHC/C among patients with liver cirrhosis in comparison with control subjects (Shibuya et al. 2003). Another study which investigated the activity and protein content of CYP3A4 in cirrhotic liver tissue revealed a significant reduction in both parameters of the enzyme in comparison with healthy liver (Yang et al. 2003). Also, the anatomical and pathophysiological changes associated with hepatic cirrhosis might contribute in the reduction of CYP3A4 metabolizing activity (Shibuya et al. 2003; Horiike et al. 2005). All these findings might explain the reduced clearance of highly extracted drugs like lidocaine in patients with liver cirrhosis so that the given dose of the drug has to be reduced (Orlando et al. 2003).

A significant reduction in the urinary ratio of 6β-OHC/C was observed in Child B cirrhotic patients who had no cholestasis while it showed a tendency to increase (but not significant) in cholestatic Child B patients in comparison with control subjects (Table 4). These findings reflect a selective influence of the cholestasis on the urinary ratio of 6β-OHC/C in Child B cirrhotic patients especially this group collectively and without stratification according to concomitant cholestasis showed no significant difference in the ratio in comparison with the control group (Table 3). It has been suggested that P450 3A hydroxyls activity and 3A protein are reduced in patients with liver cirrhosis without cholestasis (George et al. 1995). Elevation of the urinary ratio of 6β-OHC/C in the presence of cholestasis may have many reasons at molecular levels of CYP3A proteins. Bile acid or drug-induced cholestasis may lead to activation of pregnane X receptor (PXR) which induces human cytochrome P4503A4 to catalyze 6-hydroxylation of bile acids for renal excretion (Gnerre et al. 2004; Li and Chiang, 2005; Li and Chiang, 2006). Furthermore, CYP3A4 may be adaptively up-regulated in cholestasis with unknown mechanism. It is suggested that CYP3A4 can metabolize lithocholic acid into 3-dehydrolithocholic acid, a potent activator of the nuclear receptors, pregnane X receptor and 1,25-dihydroxy vitamin D3 receptor, which are known to regulate the expression of CYP3A4 (Bodin et al. 2005).

On the other hand, stratification of Child C cirrhotic patients according to cholestasis did not affect the significantly lowered values of urinary ratio of 6β-OHC/C in comparison with either control subjects or Child B cirrhotic patients (Table 4). Therefore, it seems that the severe injury of liver cell in Child C patients is more influential than other concomitant pathological findings in the liver such as cholestasis on the ratio of 6β-OHC/C.

In patients with liver cirrhosis, hypoalbuminemia is a common biochemical finding (Table 1) which represents a reduction in the synthetic power of cirrhotic liver to produce albumin. This might explain the positive correlation between serum albumin and urinary values of 6β-OHC/C in patients with liver cirrhosis (fig. 1). The impaired expression of hepatic proteins including CYP3A4 might affect the metabolism of endogenous cortisol into its metabolite; 6 β-hydroxycortisol (Shibuya et al. 2003; Horiike et al. 2005). Also, the concentration of albumin in blood is thought to affect the disposition or biotransformation of drugs by regulating their penetration into liver (Ludden et al. 1997). Therefore, hypoalbuminemia in patients with liver cirrhosis may result in alteration in the proposed protein-facilitated metabolism of drugs and endogenous substances (e.g. cholesterol & sex steroids) which are metabolized by CYP3A4 (Baba et al. 2002). The observed negative correlation (but not significant) between the urinary values of 6 β-OHC/C and both ALT and prothrombin time in our results reflected a trend of CYP3A4 activity to reduce in liver cirrhosis.

In conclusion, patients with chronic liver cirrhosis might have a reduction of metabolizing activity of CYP3A4 enzymes which could be identified by measuring the urinary ratio of 6 β-OHC/C. This reduction is more apparent in severe liver injury. Liver cirrhosis associated with cholestasis is likely to modify the activity of CYP3A4.

A prospective study is suggested on a large number of Egyptian healthy controls and patients with liver cirrhosis to study the correlation between CYP3A4 mRNA titer in blood and the urinary ratio of 6 β-hydroxycortisol/cortisol in a trial to explain the wide variability in CYP3A4 activity among Egyptians.

## Figures and Tables

**Table 1:** Characteristics of cirrhotic patients included in the study.

	Total	Reference values*
N	57 (Child B: 28)** (Child C: 29)**	
Age^1^ (years)	50 (45–60)	
Gender (F/M)	8/49	
Smoking^2^	16/57	
Ascites^2^	29/57	
Serum albumin^1^ (g/l)	23 (19.9–28.6)	25.4–36.2
Total serum bilirubin^1^ (umol/l)	44 (24.5–100)	11.0–17.0
Serum ALT^1^ (U/L)	56 (40–70)	29.0–41.0
Serum AST^1^ (U/L)	83 (60–130)	37.7–54.5
Serum ALP^1^ (U/L):		135–195
no cholestasis (n = 40)	117 (82.5–170)	
with cholestasis (n = 17)	301 (238–467)	
GGT^1^ (U/L):		5–85
no cholestasis (n = 40)	50.9 (23–86.9)	
with cholestasis (n = 17)	879.9 (698.7–1087)	
Prothrombin time^1^ (seconds)	18.8 (16–20)	11.0–15.0
Serum creatinine^1^ (umol/l)	109 (70–190)	80.4–115.8
CLcr 1,a (ml/min)	85 (60–105)	80–140
HBs Ag positive^2^	15/57 (26%)	
HCV Ab positive^2^	42/57 (74%)	
HBs Ag positive & HCV Ab positive^2^	46/57 (80%)	

ALT: alanine transferase, AST: aspartate transferase, ALP: alkaline phosphatase, GGT: gama glutamyl transpferase, CLcr: creatinine clearance.

* As followed up in the laboratory.

** Child-Pugh classification

1 Data represent the median values with the 25th and 75th percentiles in parenthesis.

2 Data represent the number of patients in this category relative to the total number of patients.

a Estimated according to Cockroft equation (Cockcroft et al. 1976).

**Table 2:** Urinary ratio of 6β-OHC/C (ng/ml) in healthy control subjects.

Control subjects	Urinary ratio of 6β-OHC/C
	Median
Total: (n = 36)	10.4
Male: (n = 26)	14.7^a^
Female: (n = 10)	3.6

a Mann-Whitney U-test (versus female; p < 0.01)

**Table 3:** Urinary ratio of 6β-OHC/C (ng/ml) in cirrhotic patients compared with healthy control subjects.

Group	Urinary ratio of 6β-OHC/C (ng/ml)	
	Median	p-value#
Control subjects (n = 36)	10.4	
Cirrhotic patients (n = 57):
Child B (n = 28; ♀: n = 3)	7.8^a^	<0.05
Child C (n = 28; ♀: n = 5)	1.3^b^	<0.001

a versus Child C,

b versus control.

# Mann-Whitney U- test.

**Table 4:** Urinary ratio of 6β-OHC/C (ng/ml) in cirrhotic patients with and without cholestasis in comparison with healthy control subjects.

6β-OHC/C	Control	Child B cirrhosis		Child C cirrhosis	
(ng/ml)	subjects (n = 36)	Choles (n = 9)	No Choles (n = 19)	Choles (n = 8)	No Choles (n = 21)
Median value	10.4	17*	6^a^,#	0.5^b^	1.9^c^

Choles = cholestasis. No Choles = no cholestasis.

Mann-Whitney U-test was used for statistical analysis.

a Versus control: P < 0.05,

b P < 0.01,

c P < 0.001

* Versus Child C with cholestasis: P < 0.001

# Versus Child C without cholestasis: P < 0.05
